# The Nuclear IκB Family Protein IκB_NS_ Influences the Susceptibility to Experimental Autoimmune Encephalomyelitis in a Murine Model

**DOI:** 10.1371/journal.pone.0110838

**Published:** 2014-10-27

**Authors:** Shuhei Kobayashi, Akira Hara, Takayuki Isagawa, Ichiro Manabe, Kiyoshi Takeda, Takashi MaruYama

**Affiliations:** 1 Laboratory of Cell Recognition and Response, Tohoku University, Miyagi, Japan; 2 Department of Pathology, Gifu University Graduate School of Medicine, Gifu, Japan; 3 Department of Genomic Pathology, Tokyo Medical and Dental University, Tokyo, Japan; 4 Department of Cardiovascular Medicine, The University of Tokyo, Tokyo, Japan; 5 Laboratory of Immune Regulation, Osaka University, Osaka, Japan; 6 Department of Cell signaling, Gifu University Graduate School of Medicine, Gifu, Japan; University of Nebraska-Lincoln, United States of America

## Abstract

The nuclear IκB family protein IκB_NS_ is expressed in T cells and plays an important role in Interferon (IFN)-γ and Interleukin (IL)-2 production. IκB-ζ, the most similar homolog of IκB_NS_, plays an important role in the generation of T helper (Th)17 cells in cooperation with RORγt, a master regulator of Th17 cells. Thus, IκB-ζ deficient mice are resistant to Th17-dependent experimental autoimmune encephalomyelitis (EAE). However, IκB-ζ deficient mice develop the autoimmune-like Sjögren syndrome with aging. Here we found that IκB_NS_-deficient (*Nfkbid^−/−^*) mice show resistance against developing Th17-dependent EAE. We found that *Nfkbid^−/−^* T cells have decreased expression of IL-17-related genes and RORγt in response to Transforming Growth Factor (TGF)-β1 and IL-6 stimulation. Thus, IκB_NS_ plays a pivotal role in the generation of Th17 cells and in the control of Th17-dependent EAE.

## Introduction

The transcriptional regulator IκB_NS_ is a member of the nuclear IκB family, which also includes IκB-ζ and Bcl-3. IκB_NS_ expression occurs in T cells and is rapidly induced upon T cell receptor (TCR) stimulation [1]. IκB_NS_ has 7 ankyrin repeat domains that bind the p50 subunit of the DNA-binding protein nuclear factor-kappa B (NF-κB), but has no DNA-binding domain [2]. IκB_NS_ interacts with NF-κB to control Interleukin (IL)-6 gene expression in macrophages [3], [4]. In T cells, IκB_NS_ positively regulates IL-2 expression, a target of NF-κB [5]. Previously, Schmitz's group showed that IκB_NS_ intrinsically induces Forkhead box P3 (Foxp3) positive regulatory T cells (Tregs) *in vivo* and *in vitro* [6]. Foxp3 is a master regulator of Tregs and can be induced by NF-κB activation [7].

TCR and Transforming Growth Factor (TGF)-β signaling are necessary for the generation of both Tregs and T helper (Th)17 cells [8]. IL-17 is a proinflammatory cytokine that plays an important role in autoimmune diseases and against bacterial infections [9]. The nuclear IκB family protein IκB-ζ can be induced in T cells in response to TGF-β1 and IL-6 stimulation and positively regulates Th17 generation in cooperation with RORγt [10]. Therefore, IκB-ζ-deficient mice are resistant to Th17-dependent experimental autoimmune encephalomyelitis (EAE), a model of multiple sclerosis. However, these mice have more effecter memory T cells and produce more Interferon (IFN)- γ in the periphery, leading to a Sjögren-like syndrome with age [11].

Here, we demonstrate that IκB_NS_-deficient (*Nfkbid^−/−^*) mice are resistant to Th17-dependent EAE. Further analysis revealed that the percentage of Th17 cells in the draining lymph nodes (LNs) of myelin oligodendrocyte glycoprotein (MOG)-immunized *Nfkbid^−/−^* mice was decreased relative to that of control mice. In addition, IκB_NS_-deficient T cells were less capable of generating Th17 cells in response to TGF-β1 and IL-6. Mechanistically, we found that IκB_NS_–deficient T cells show decreased RORγt induction in response to TGF-β1 and IL-6.

## Materials and Methods

### Mice

*Nfkbid^−^* ^/*−*^ mice (having a mixed C57/BL6 × BALB/c genetic background) were established as described previously [3]. All mice were maintained in specific pathogen-free conditions in the animal facilities of Tohoku University. All animal protocols were approved by the Institutional Committee for the Use and Care of Laboratory Animals of Tohoku University (2013MA029, 2013MA031 and 2013MA032).

### Cells

CD4^+^CD25^−^ T cells were prepared from mouse spleens using a CD4^+^CD25^+^ isolation kit II (Miltenyi Biotec; Bergisch Gladbach, Germany).

### EAE induction and analysis of cytokine production

*Nfkbid* ^+/+^ and *Nfkbid* ^−/−^ mice were injected subcutaneously (on the lower back) on day 0 with emulsions containing complete Freund's adjuvant (CFA; BD Difco™; Detroit, MI), 100 µg MOG peptide (MEVGWYRSPFSRVVHLYRNGK; MBL International Corporation, Nagoya, Japan), and 0.5 mg *Mycobacterium tuberculosis* H37RA (BD Difco™). In addition, these mice received 500 ng pertussis toxin (Sigma) by i.p. injection to boost immunological responses on day 0 and 2. These mice were observed until day 21 after immunization and clinical signs of EAE were scored according to a previously described protocol [12]. To study cytokine production, draining lymph node cells were derived and cultured for 72 h in the presence of 10 ng/ml MOG peptide.

### *In vitro* T cell culture

Purified CD4^+^CD25^−^ T cells were cultured in RPMI 1640 medium containing 10% heat-inactivated fetal calf serum, 100 units/mL penicillin and 100 µg/mL streptomycin at 37°C in 5% CO_2_. For cells in the Th0 condition, anti-CD3 (1 µg/mL) + anti-CD28 (1 µg/mL) stimulation was used. For cells in the Th17 condition, anti-CD3 (1 µg/mL) + anti-CD28 (1 µg/mL) with TGF-β1 (2 ng/mL) and IL-6 (50 ng/mL) stimulation was used [13].

### Plasmids, antibodies, and cytokines

Expression vectors encoding FLAG-tagged mouse RORγt and IκB-ζ were constructed as described previously [10], [14]. Mouse IκB_NS_ was inserted into a pcDNA3-FLAG vector at the *EcoR*I and *BamH*I sites. The mouse IL-17A promoter (−6647 to +1) was inserted into a pGL4.12 vector at the *Nhe*I and *Hin*dIII sites. pGL4 and pcDNA3 were obtained from Life Technologies (Rockville, MD), and phRL-TK was obtained from Promega Corp. (Madison, WI).

APC-conjugated anti-mouse CD62L (MEL-14), APC-conjugated anti-mouse IL-17A (TC11-18H10.1), PerCP/Cy5.5-conjugated anti-mouse CD8α (53-6.7), and Pacific Blue-conjugated anti-mouse CD4 (GK1.5) antibodies were purchased from BioLegend, Inc. (San Diego, CA). PE-conjugated anti-mouse IFN-γ (XMG1.7), purified anti-mouse CD3 (145-2C11), and purified anti-mouse CD28 (37.51) antibodies were obtained from eBioscience, Inc. (San Diego, CA). Rat anti-mouse Galectin-3 was obtained from Bay bioscience Co., Ltd (Hyogo, Japan). Biotinylated anti-rat IgG (E0468) was obtained from DAKO (Glostrup, Denmark). Recombinant human TGF-β1 and mouse IL-6 were obtained from PeproTech, Inc. (Rocky Hill, NJ).

### Flow cytometric analysis

Cell surface antigens were stained in the dark at 4°C with antibodies diluted in PBS containing 0.5% BSA (FACS buffer). To study intracellular cytokine production, cells were stimulated with 250 nM ionomycin (BD Bioscience, San Jose, CA) and 50 nM phorbol 12-myristate 13-acetate (Sigma-Aldrich) in the presence of GolgiStop (BD Bioscience) for 4 h at 37°C in 5% CO_2_. Cells were fixed with 4% paraformaldehyde-PBS, permeabilized with 0.5% saponin in FACS buffer, and then stained in the dark at 4°C with the indicated antibodies [11]. Stained cells were analyzed with a Gallios™ flow cytometer (Beckman Coulter, Inc.; Brea, CA) and the data obtained were analyzed with FlowJo software (Tree Star, Inc.; Ashland, OR).

### Enzyme-linked immunosorbent assays (ELISAs)

ELISA kits for IL-17A and IFN-γ (eBioscience) were used to quantify the respective cytokines in culture supernatants, according to the manufacturer's protocols.

### Chromatin immunoprecipitation (ChIP)

Cultured CD4^+^ T cells were fixed in 1% formaldehyde, exposed to 0.2 M glycine to halt the fixation process, and washed in ice-cold PBS containing 0.5% BSA. Subsequently, cells were lysed by sonication in SDS lysis buffer containing 1% (wt/vol) SDS, 10 mM EDTA, and 50 mM Tris (pH 8.0). Cellular debris was removed by centrifugation. A ChIP assay was performed using antibodies against acetyl-histone H3 (Lys27) and normal rabbit IgG (Cell Signaling Technology; Danvers, MA) and Dynabeads Protein G (Life Technologies). Immunoprecipitated and input DNA was then analyzed by quantitative PCR using SYBR Premix EX Taq (Takara Bio). The sequences of primers used for quantitative PCR are as follows: 5′-GCTGCTGTTTCCTTGAGAGG-3′ and 5′-GCTGGATAAGGCAGGAACAG-3′ for conserved non-coding sequence (CNS) 1; 5′- CTGAGTTGGGGGCTGTGTAT -3′ and 5′-CATATCGAGGGTGTCGGACT-3′ for CNS 2; 5′-CAGCAGACACACATGCAAGA-3′ and 5′-CCTCAGGGGAGGGAATTAAG-3′ for CNS 3; 5′-CACCTCACACGAGGCACAAG-3′ and 5′-ATGTTTGCGCGTCCTGATC-3′ for the *Il-17a* promoter; and 5′-CACTTCCTGAAGGGGAATCA-3′ and 5′-GGGTGGGCTTAGAAGAGAGG-3′ for the *Il-17f* promoter.

### Histology

Tissues were fixed by immersion in 10% formalin in phosphate-buffered saline and embedded in paraffin blocks. Three-micrometer-thick sections were stained with hematoxylin and eosin (HE staining) or luxol fast blue solution and cresyl violet solution (Klüver-Barrera staining), and then examined by light microscopy. Immunohistochemistry of galectin-3 was described previously [15]. Briefly, the paraffinized sections were antigen revealed by using a 0.01 M citrate buffer (pH 6.0) by the PascalR heat-induced target retrieval system (DAKO). Anti-galectin-3 antibody using at a dilution of 1∶100 in 2% BSA/PBS were added on the slides and incubated overnight at 4°C. Anti-galectin-3 antibody was detected with a biotinylated anti-Rat IgG (1∶200) for 30 min, followed by incubation with avidin-coupled peroxidase (Vectastain ABC kit, Vector Laboratories) for 30 min. The peroxidase binding sites were detected by staining with 3,3′-diaminobenzidine (DAB) in 50 mM Tris–EDTA buffer, pH 7.6.

### Luciferase Assays

HEK 293 cells (1×10^5^ cells) were transfected using the calcium phosphate-DNA coprecipitation method with IL-17A reporter and expression vectors (pcDNA3-RORγt, IκB_NS_, and IκB-ζ) with pRL-TK-Luc. Twenty-four hours after transfection, the medium was changed and the cells were incubated for a further 24 h. Luciferase activities were measured using the Dual-Luciferase Reporter Assay System, according to the manufacturer's instructions (Promega Corp., Madison, WI). Data shown are the mean ± SE of duplicate samples from a representative of at least 3 independent experiments.

### Statistical Analysis

Paired data were evaluated using the Student's t test.

## Results

### *Nfkbid* ^−/−^ mice maintain immune homeostasis

**Figure 1:**
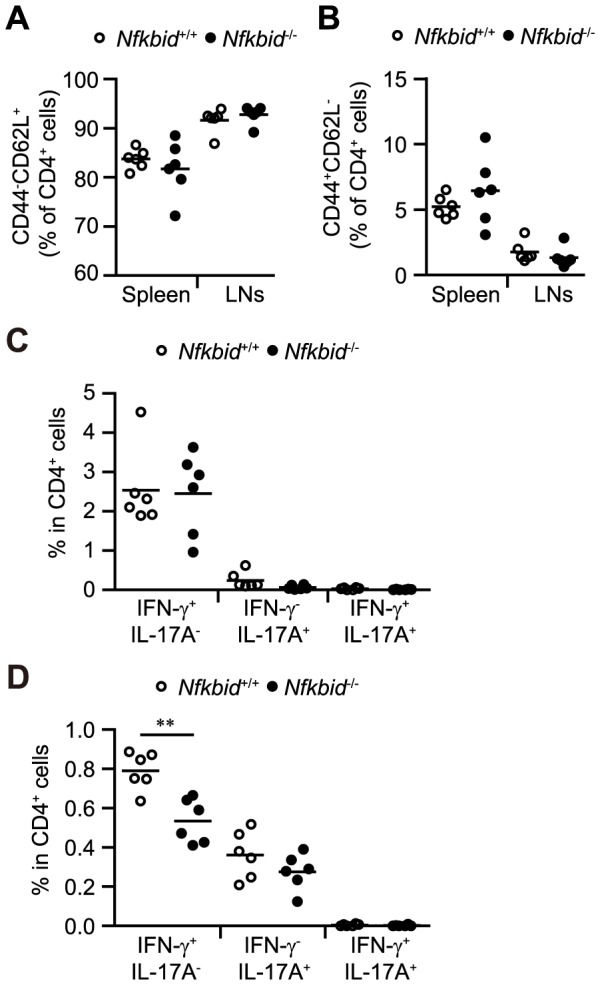
Characteristics of immune homeostasis in *Nfkbiz* ^−/−^ mice. (A, B) Naive CD4^+^ cells in the spleen and lymph nodes (LNs) of 8–12 week old *Nfkbid* ^+/+^ and *Nfkbiz* ^−/−^ mice. (C, D) Flow cytometric analysis of IFN-γ- and IL-17-producing CD4^+^ cells isolated from the spleen (C) and LNs (D) of *Nfkbid* ^+/+^ and *Nfkbiz* ^−/−^ mice at 8–12 weeks of age. Paired data were evaluated using the Student's t test. **p<0.01.

A previous study showed that IκB_NS_ plays an important role in the generation of Tregs [6]. However, *Nfkbid* ^−/−^ mice and *bumble* mutant mice (harboring a stop codon after exon 4 of the *Nfkbid* gene) appear healthy and do not exhibit an inflammatory phenotype in the periphery [16]. IκB_NS_ shares greatest homology with IκB-ζ (43% identity at the gene level). We confirmed that *Nfkbid* ^−/−^ mice appear healthy and can live for up to 6 months without disease manifestation. At 8–12 weeks old, the percentage of effector/memory and naïve CD4^+^ cells in the peripheral LNs and spleen were comparable in *Nfkbid* ^+/+^ and *Nfkbid* ^−/−^ mice (Fig. 1A and 1B). Although the overall percentages of IFN-γ- and IL-17A-producing CD4^+^ cells in the spleen and peripheral LNs were low (Fig. 1C and 1D), we were still able to demonstrate that the percent of IFN-γ-producing CD4^+^ cells in the peripheral LNs was lower in *Nfkbid* ^−/−^ mice compared to *Nfkbid* ^+/+^ mice (Fig. 1D).

### *Nfkbid* ^−/−^ mice resist EAE development

**Figure 2:**
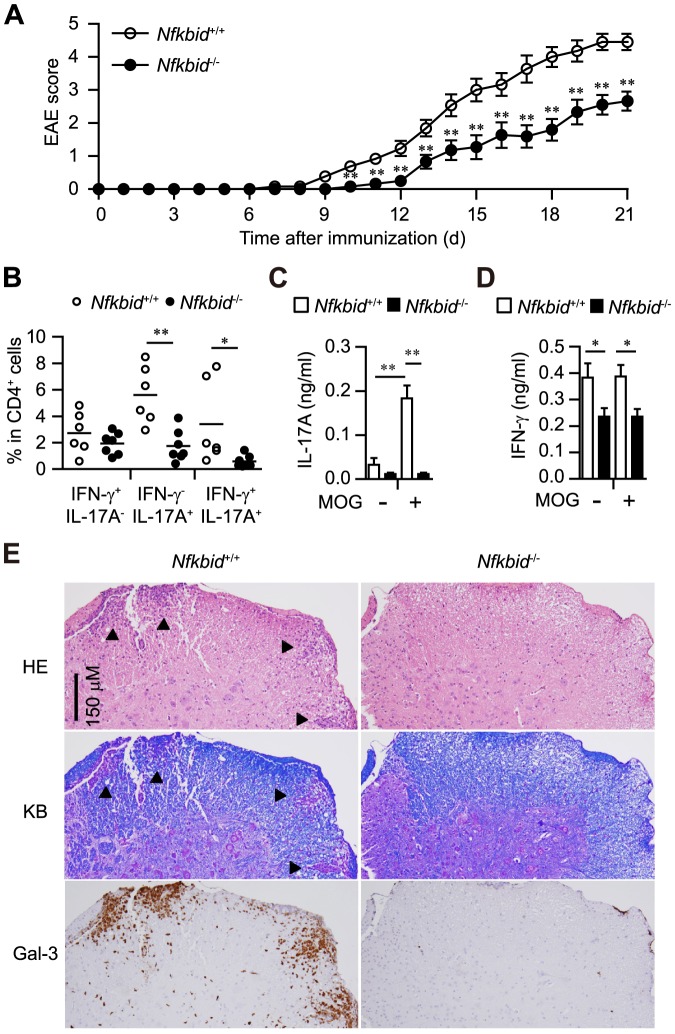
Experimental autoimmune encephalomyelitis (EAE) model in *Nfkbiz* ^−/−^ mice. (A) Disease progression of EAE in *Nfkbiz* ^+/+^ (n = 11–13) and *Nfkbiz* ^−/−^ mice (n = 9–11). (B–D) Analysis of mice 12 days after immunization. (B) Cytokine profile of CD4^+^ cells in draining LNs. (C, D) Measurement of IL-17A (C) and IFN-γ (D) supernatant concentrations by ELISAs (*Nfkbiz* ^+/+^: n = 5; *Nfkbiz* ^−/−^: n = 6), using cultured draining LNs incubated in the presence or absence of MOG peptide (10 ng/ml) for 72 h. Data shown represent mean ± S.E. Paired data were evaluated using the Student's t test. *p<0.05, **p<0.01. (E) Histology of spinal cord specimens in EAE models. Twelve days after MOG immunization, *Nfkbiz* ^+/+^ and *Nfkbiz* ^−/−^ mice were sacrificed and their lumber section of spinal codes were collected. Three-micrometer-thick sections were stained with hematoxylin and eosin (HE), Klüver-Barrera staining (KB) or galectin-3 (Gal-3) immunohistochemistry. Serial sections were used for HE staining, KB staining and Gal-3 immunohistochemistry. Arrowheads in HE staining and KB staining indicate the demyelinated lesions. Data are representative of 3 independent experiments.

Next, we generated classical Th17-dependent EAE models by immunizing mice with the MOG peptide [17], [18]. Ten to twelve days after MOG immunization, *Nfkbid* ^+/+^ mice developed EAE, starting with paralysis of the tail, followed by paralysis in the front and hind limbs between days 18 and 21 (Fig. 2A). However, *Nfkbid* ^−/−^ mice showed significantly fewer clinical signs of diseases (Fig. 2A). Further, *Nfkbid* ^−/−^ mice have fewer IL-17A-producing CD4^+^ cells and reduced MOG antigen-specific IL-17A production in their draining LNs (Fig. 2B–D). We also confirmed that lymphocyte infiltration and demyelination occurred in the lumber section of spinal cords of *Nfkbid* ^+/+^ EAE model mice, but not in those of *Nfkbid* ^−/−^ EAE models (Fig. 2E). Galectin-3, an activation maker of monocyte/macrophages/microglia [19], was observed as dark-brown staining in injured white matter of spinal cord in EAE model. We found that many galectin-3 positive cells in the lumber section of spinal cords of *Nfkbid* ^+/+^ EAE model mice, but not *Nfkbid* ^−/−^ EAE model mice (Fig. 2E). In addition, adoptive transfer of CD4^+^ T cells from EAE models of *Nfkbid* ^−/−^ mice to naïve *Nfkbid* ^+/+^ mice caused tail and hind limb paralysis, although these symptoms were less severe than those observed with adoptive transfer of CD4^+^ T cells from EAE models of *Nfkbid* ^+/+^ mice (Fig. S1). Thus, T cells serve an intrinsic role in the resistance of EAE in *Nfkbid* ^−/−^ mice.

### Reduced Th17 cell differentiation in *Nfkbid* ^−/−^ T cells

**Figure 3:**
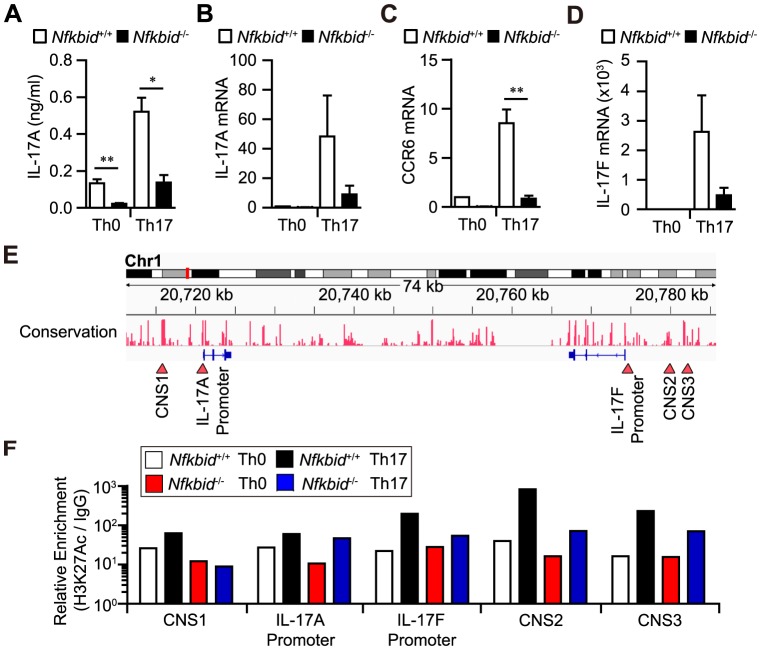
*Nfkbiz* ^−/−^ mouse T cells fail to generate Th17 cells *in vitro*. (A–D) Expression of IL-17A protein or *Il-17a* mRNA (A, B) and of the Th17-related mRNAs *Ccr6* and *Il-17f* (C, D) in CD4^+^ T cells from *Nfkbiz* ^+/+^ and *Nfkbiz* ^−/−^ mice, cultured for 48 h under Th0 or Th17 conditions. (E) Diagram of *Il-17a* and *Il-17f* gene conservation. Red-arrows indicate the *Il-17a* promoter, the *Il-17f* promoter, and the CNS 1, CNS 2, and CNS 3 regions. (F) ChIP analysis of H3K27Ac. Cells were cultured under Th0 or Th17 conditions for 48 h. Data shown are from one experiment that was representative at three independent experiments. (A–D) Data shown represent mean ± S.E. (n = 3). Paired data were evaluated using the Student's t test. *p<0.05, **p<0.01.

Next, we examined whether *Nfkbid* ^−/−^ T cells are capable of differentiating into Th17 cells. We found that *Nfkbid* ^−/−^ T cells show decreased expression of IL-17A (Fig. 3A and 3B) and Th17-related genes (*Ccr6* and *Il-17f*; Fig. 3C and 3D) in response to TGF-β1 and IL-6 stimulation. In addition, detection of Acetylated histone H3 on Lysine 27 (H3K27Ac), a histone modification associated with open chromatin configurations, was reduced in the conserved non-coding sequence (CNS) 1, CNS 2, CNS 3, and *Il-17f* promoter regions in *Nfkbid* ^−/−^ T cells under Th17 conditions compared to that observed in *Nfkbid* ^+/+^ T cells (Fig. 3E–F). These regions potentially regulate *Il-17* gene expression [20]. Interestingly, the acetylation status of the CNS 1 region in *Nfkbid* ^−/−^ T cells did not change under Th17 conditions compared with Th0 conditions (Fig. 3F). A previous study indicated that the CNS 1 region plays an important role for both IL-17A and IL-17F expression [21]. Thus, *Nfkbid* ^−/−^ T cells were impaired in generating Th17 cells in response to TGF-β1 and IL-6 because of reduced histone H3 acetylation in the CNS 1 regions.

### IκB_NS_ does not control *il17a* gene expression

**Figure 4:**
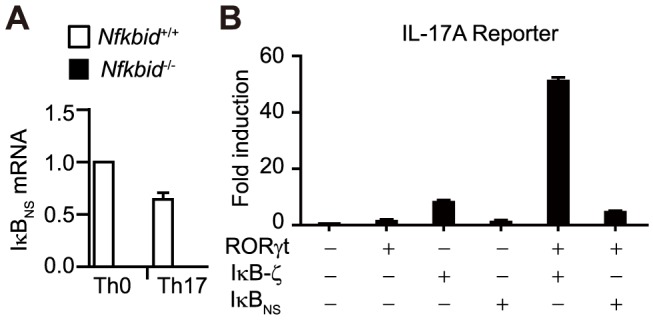
IκB_NS_ does not regulate *Il-17a* gene expression. (A) Relative IκB_NS_ mRNA expression levels in cultured CD4^+^ T cells under Th0 or Th17 conditions. IκB_NS_ mRNA expression in CD4^+^ T cells cultured under Th0 conditions from control mice was set to a value of 1. Data shown represent mean ± S.E. (n = 3) (B) *Il-17a* promoter activity presented as the fold-increase over that observed in HEK293 cells transfected with the empty vector. Data shown are the mean ± S.D. of duplicate samples and are representative of three independent experiments.

IκB-ζ, a homolog of IκB_NS_, can be upregulated in T cells in response to TGF-β1 and IL-6 stimulation, directly binds to CNS1, and positively regulates IL-17A expression in cooperation with RORγt [22], [23]. Here, we found that IκB_NS_ expression was comparable under Th0 and Th17 conditions (Fig. 4A). In addition, IκB_NS_ expression had no effect on IL-17A reporter activity, even in the presence of RORγt (Fig. 4B). Thus, while IL-17A expression may be regulated by IκB-ζ, the homolog IκB_NS_ does not transcriptionally control IL-17A.

### Reduced RORγt expression in *Nfkbid* ^−/−^ T cells

IκB_NS_ can be induced upon TCR stimulation and can control NF-κB transcriptional activity [6], [24]. In T cells, IκB_NS_ deficiency leads to decreased production of IL-2 (a target of NF-κB) in response to TCR stimulation [5]. Thus, *Nfkbid* ^−/−^ T cells have a reduced ability to activate NF-κB in response to TCR stimulation. Rel (NF-κB subunit)-deficient T cells fail to generate Th17 cells since RORγt induction is diminished in response to TGF-β1 and IL-6 stimulation [25].

**Figure 5:**
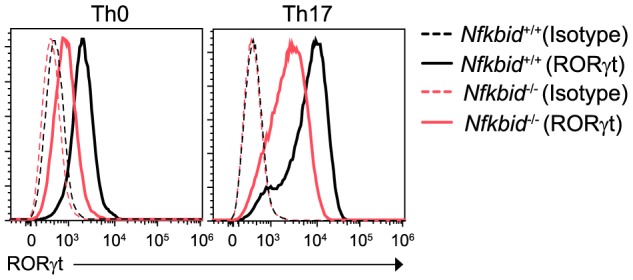
*Nfkbiz* ^−/−^ T cells show decreased RORγt expression. RORγt expression in CD4^+^ T cells from *Nfkbiz* ^+/+^ and *Nfkbiz* ^−/−^ mice, cultured for 72 h under Th0 or Th17 conditions. Data are representative of three independent experiments.

To understand the molecular mechanism underlying the control of IL-17A gene expression by IκB_NS_, we examined the expression of RORγt (a master regulator of Th17) and found that it was decreased in *Nfkbid* ^−/−^ T cells (Fig. 5). Thus, IκB_NS_ controls NF-κB activation, which plays a pivotal role in RORγt expression and Th17 cell differentiation.

## Discussion

IκB_NS_, a member of the nuclear IκB family of proteins, is induced by TCR stimulation in thymocytes [1], [26]. Interestingly, *Nfkbid* ^−/−^ mice show a high sensitivity to lipopolysaccharide (LPS)-induced endotoxin shock and 4,4-dimethyl-4-silapentane-1-sulfonic acid-induced colitis because *Nfkbid* ^−/−^ DCs and macrophages produce large amounts of IL-6 in response to LPS stimulation [3]. It is well known that IL-6 positively regulates Th17 cell generation [27]. In addition, IκB_NS_ may play a pivotal role in IL-10 production from regulatory DCs [28]. Thus, the intrinsic role of IκB_NS_ in T cells may contribute to exacerbating Th17-dependent EAE. Although IκB_NS_ is important for Foxp3^+^Tregs generation [6], *Nfkbid* ^−/−^ mice and *bumble* mutant mice appear healthy and do not exhibit an inflammatory phenotype in the periphery (Fig. 1) [16]. IκB_NS_ may play a minor role in the immune suppression ability of Tregs.

IκB-ζ, a homolog of IκB_NS_, has a transcriptional activation domain and is important for Th17 cell differentiation [10]. IκB_NS_ does not have a similar homologous transcriptional activation domain. In addition, IκB-ζ expression in T cells is dependent on TGF-β1 and IL-6 stimulation [10]. Thus, the regulation of IL-17A gene expression by IκB-ζ and IκB_NS_ proceeds by different mechanisms.

*Nfkbid* ^−/−^ T cells show reduced proliferation and NF-κB activation in response to TCR [5]. However, a previous study showed that IL-17A promoter activity is dispensable for NF-κB activation [25]. Our ChIP data revealed that acetylation of histone H3 in the CNS 1 region does not change in *Nfkbid* ^−/−^ T cells in response to TGF-β1 + IL-6 (Fig. 3F). The CNS 1 region normally controls both IL-17A and IL-17F gene expression [21]. RORγt, a master regulator of Th17, has the ability to bind both CNS 1 and the *Il-17a* promoter region, and it positively regulates IL-17A gene expression [29]. Therefore, our results indicate that *Nfkbid* ^−/−^ T cells showed impaired Th17 cells differentiation because of a reduction in RORγt expression and histone H3 acetylation in the CNS 1 region. In conclusion, we show that IκB_NS_ deficiency causes resistance to Th17-dependent autoimmune disease.

## Supporting Information

Figure S1 **Passive EAE model using adoptive T cell transfer.** Collected draining LNs from the *Nfkbiz* ^+/+^ and *Nfkbiz* ^−/−^ mice at day 12 after MOG immunizations. LN cells were re-stimulated by MOG (10 ng/ml) after 3 days in culture, and CD4^+^ T cells were isolated using the CD4^+^CD25^+^ Regulatory T cell Isolation Kit (Miltenyi Biotec). *Nfkbiz* ^+/+^ mice (n  =  3–4/group) were intravenously injected (5 × 10^5^ CD4^+^ T cells/mouse) and EAE symptoms were scored for up to 12 days. In addition, these mice received 500 ng pertussis toxin (Sigma) by i.p. injection to boost their immunological responses on Days 0 and 2. Data shown represent mean + S.E. Paired data were evaluated using the Student's t test. *p <0.05.
(EPS)
