# Correction: The Nuclear IκB Family Protein IκB_NS_ Influences the Susceptibility to Experimental Autoimmune Encephalomyelitis in a Murine Model

**DOI:** 10.1371/journal.pone.0118159

**Published:** 2015-02-06

**Authors:** The PLOS ONE Staff

The gene name *Nfkbiz^−/−^* appears incorrectly in the figure legends. The correct gene name should be *Nfkbid^−/−^*.

The authors have provided the corrected figure legends below.

**Fig 1:**
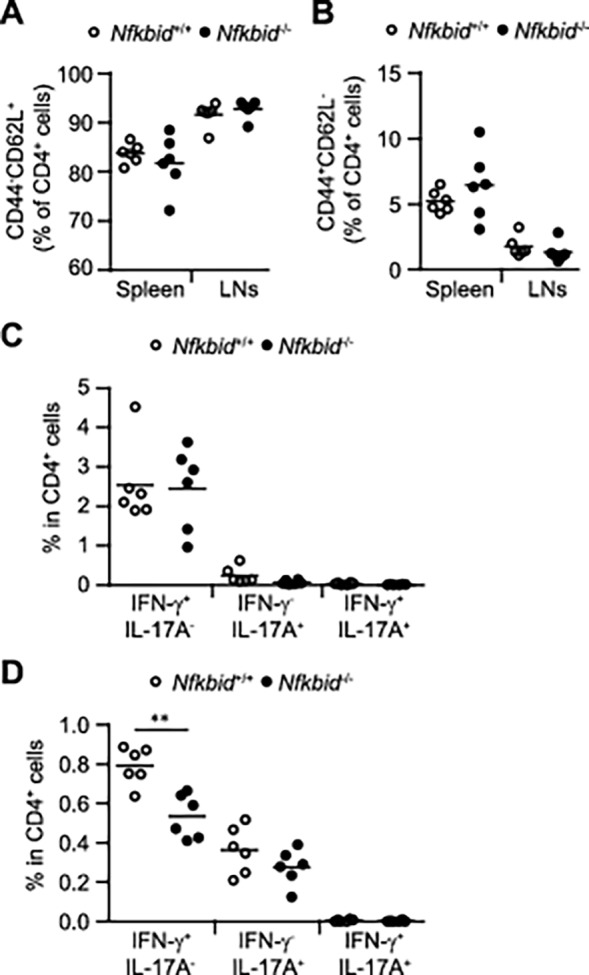
Characteristics of immune homeostasis in *Nfkbid* ^−/−^ mice. (A, B) Naive CD4^+^ cells in the spleen and lymph nodes (LNs) of 8–12 week old *Nfkbid* ^+/+^ and *Nfkbid* ^−/−^ mice. (C, D) Flow cytometric analysis of IFN-γ- and IL-17-producing CD4^+^ cells isolated from the spleen (C) and LNs (D) of *Nfkbid* ^+/+^ and *Nfkbid* ^−/−^ mice at 8–12 weeks of age. Paired data were evaluated using the Student’s t test. **p<0.01.

**Fig 2:**
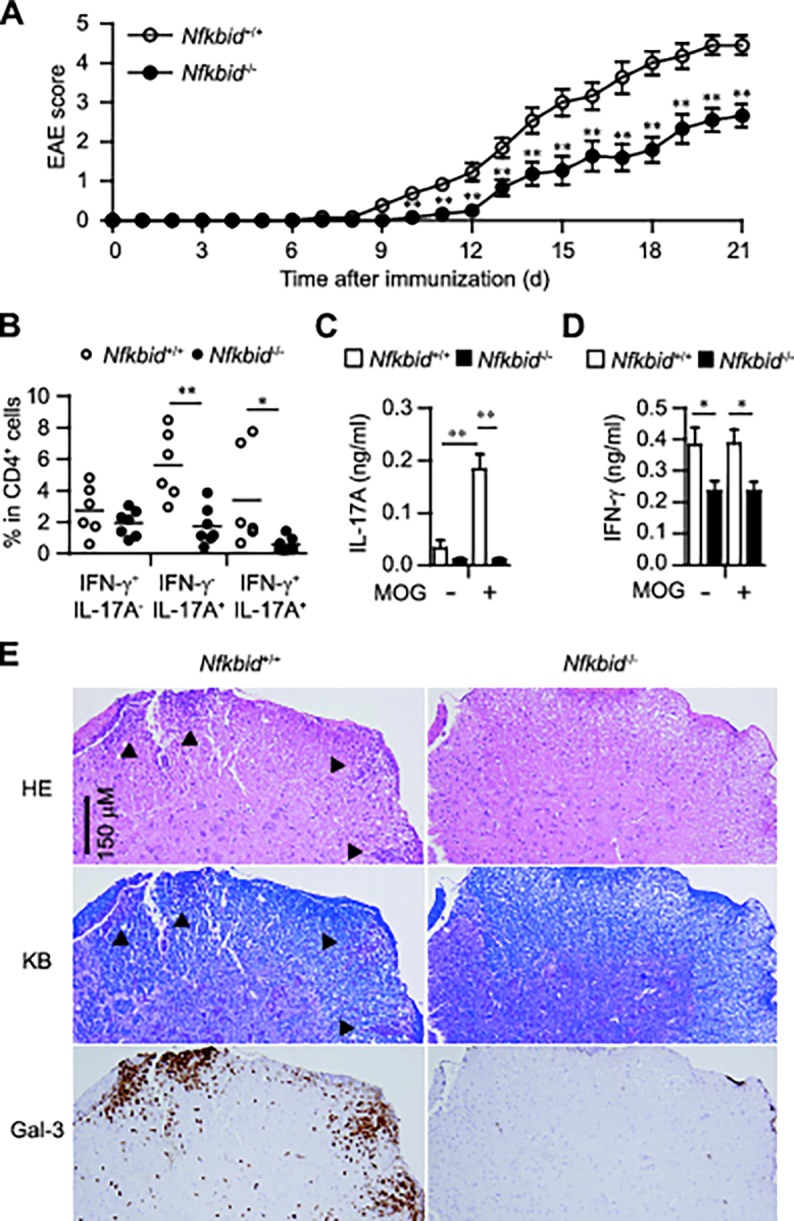
Experimental autoimmune encephalomyelitis (EAE) model in *Nfkbid* ^−/−^ mice. (A) Disease progression of EAE in *Nfkbid* ^+/+^ (n = 11–13) and *Nfkbid* ^−/−^ mice (n = 9–11). (B-D) Analysis of mice 12 days after immunization. (B) Cytokine profile of CD4^+^ cells in draining LNs. (C, D) Measurement of IL-17A (C) and IFN-γ (D) supernatant concentrations by ELISAs (*Nfkbid* ^+/+^: n = 5; *Nfkbid* ^−/−^: n = 6), using cultured draining LNs incubated in the presence or absence of MOG peptide (10 ng/ml) for 72 h. Data shown represent mean ± S.E. Paired data were evaluated using the Student’s t test. *p<0.05, **p<0.01. (E) Histology of spinal cord specimens in EAE models. Twelve days after MOG immunization, *Nfkbid* ^+/+^ and *Nfkbid* ^−/−^ mice were sacrificed and their lumber section of spinal codes were collected. Three-micrometer-thick sections were stained with hematoxylin and eosin (HE), Klüver-Barrera staining (KB) or galectin-3 (Gal-3) immunohistochemistry. Serial sections were used for HE staining, KB staining and Gal-3 immunohistochemistry. Arrowheads in HE staining and KB staining indicate the demyelinated lesions. Data are representative of 3 independent experiments.

**Fig 3:**
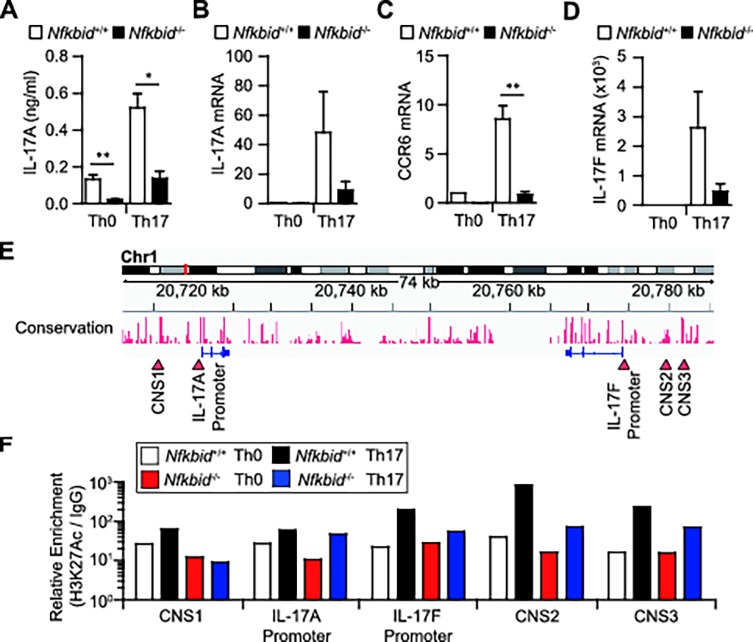
*Nfkbid* ^−/−^ mouse T cells fail to generate Th17 cells *in vitro*. (A–D) Expression of IL-17A protein or *Il-17a* mRNA (A, B) and of the Th17-related mRNAs *Ccr6* and *Il-17f* (C, D) in CD4^+^ T cells from *Nfkbid* ^+/+^ and *Nfkbid* ^−/−^ mice, cultured for 48 h under Th0 or Th17 conditions. (E) Diagram of *Il-17a* and *Il-17f* gene conservation. Red-arrows indicate the *Il-17a* promoter, the *Il-17f* promoter, and the CNS 1, CNS 2, and CNS 3 regions. (F) ChIP analysis of H3K27Ac. Cells were cultured under Th0 or Th17 conditions for 48 h. Data shown are from one experiment that was representative at three independent experiments. (A-D) Data shown represent mean ± S.E. (n = 3). Paired data were evaluated using the Student’s t test. *p<0.05, **p<0.01.

**Fig 5:**
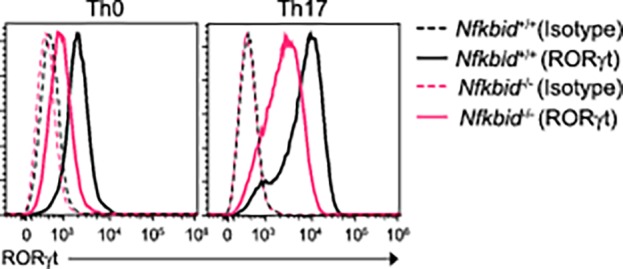
*Nfkbid* ^−/−^ T cells show decreased RORγt expression. RORγt expression in CD4^+^ T cells from *Nfkbiz* ^+/+^ and *Nfkbid* ^−/−^ mice, cultured for 72 h under Th0 or Th17 conditions. Data are representative of three independent experiments.

## Supporting Information

S1 Fig Passive EAE model using adoptive T cell transfer. Collected draining LNs from the *Nfkbid* ^+/+^ and *Nfkbid* ^−/−^ mice at day 12 after MOG immunizations. LN cells were re-stimulated by MOG (10 ng/ml) after 3 days in culture, and CD4^+^ T cells were isolated using the CD4^+^CD25^+^ Regulatory T cell Isolation Kit (Miltenyi Biotec). *Nfkbid* ^+/+^ mice (n = 3–4/group) were intravenously injected (5 × 10^5^ CD4^+^ T cells/mouse) and EAE symptoms were scored for up to 12 days. In addition, these mice received 500 ng pertussis toxin (Sigma) by i.p. injection to boost their immunological responses on Days 0 and 2. Data shown represent mean + S.E. Paired data were evaluated using the Student’s t test. *p <0.05.
(TIF)
