# Supplementary figures and images for: Correction: The Nuclear IκB Family Protein IκBNS Influences the Susceptibility to Experimental Autoimmune Encephalomyelitis in a Murine Model
Source: PLoS One. 2015 Feb 6;10(2):e0118159. doi: 10.1371/journal.pone.0118159 (PMC4319850; doi:10.1371/journal.pone.0118159)

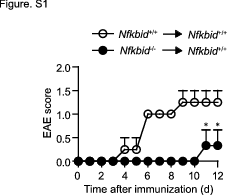

Supplement: S1 Fig — Collected draining LNs from the Nfkbid +/+ and Nfkbid −/− mice at day 12 after MOG immunizations. LN cells were re-stimulated by MOG (10 ng/ml) after 3 days in culture, and CD4+ T cells were isolated using the CD4+CD25+ Regulatory T cell Isolation Kit (Miltenyi Biotec). Nfkbid +/+ mice (n = 3–4/group) were intravenously injected (5 × 10^5^ CD4+ T cells/mouse) and EAE symptoms were scored for up to 12 days. In addition, these mice received 500 ng pertussis toxin (Sigma) by i.p. injection to boost their immunological responses on Days 0 and 2. Data shown represent mean + S.E. Paired data were evaluated using the Student’s t test. *p <0.05. (TIF) [file pone.0118159.s001.tif]
